# Fatal pedestrian injury secondary to rupture of a right coronary artery aneurysm: A case report

**DOI:** 10.1016/j.tcr.2026.101437

**Published:** 2026-09-16

**Authors:** Yoichi Hisata, Shunsuke Taguchi, Akihiko Yamamoto, Takafumi Yamada

**Affiliations:** a Division of Cardiovascular Surgery, Oita Prefectural Hospital, 2-8-1 Bunyo, Oita City, Oita, 870-8511, Japan; b Division of Emergency Medicine, Oita Prefectural Hospital, 2-8-1 Bunyo, Oita City, Oita, 870-8511, Japan

**Keywords:** Coronary artery aneurysm rupture, Acute myocardial infarction, Blunt trauma

## Abstract

**Introduction:**

Coronary artery aneurysm (CAA) rupture is a rare but life-threatening condition that typically presents as acute coronary syndrome or with hemodynamic collapse. Distinguishing whether severe trauma is the primary event or secondary to a preceding medical catastrophe poses a significant diagnostic challenge in the emergency setting.

**Case presentation:**

An 85-year-old woman, found crouched on a street, was struck by a truck and was transported to the emergency department. On arrival, she was unconscious but hemodynamically stable. Contrast-enhanced computed tomography revealed a ruptured right coronary artery aneurysm with active extravasation and surrounding hematoma, along with multiple severe traumatic injuries, including subarachnoid hemorrhage and hemopneumothorax. The patient died several hours after aggressive resuscitation.

**Conclusion:**

This case illustrates an extremely rare scenario where aneurysmal rupture likely preceded the traumatic event, leading to secondary blunt trauma. This emphasizes the critical importance of considering primary vascular emergencies as underlying causes in trauma patients who present with atypical findings.

## Highlights

• First reported case of fatal blunt trauma secondary to coronary artery aneurysm rupture
• Ruptured right coronary artery aneurysm identified on trauma screening CT
• Cardiac biomarker elevation suggested a primary cardiovascular event
• External trauma masked an underlying catastrophic vascular emergency

## Introduction

Coronary artery aneurysm (CAA) rupture is a rare but life-threatening cardiovascular event, typically presenting as acute coronary syndrome or with hemodynamic collapse. Although aneurysmal dilatation has been reported in approximately 0.3%–5% of patients undergoing coronary angiography, the incidence of complications, such as rupture, remains unclear [1].

In emergency settings, it can be challenging to distinguish whether severe trauma is the primary cause of hemodynamic collapse or a secondary event following an internal medical catastrophe.

We report a rare case of fatal pedestrian trauma that was likely secondary to the rupture of a right coronary artery aneurysm (RCA). This case illustrates the critical importance of actively considering primary vascular emergencies in trauma patients who present with atypical findings. To the best of our knowledge, the presentation of CAA rupture leading to subsequent major blunt trauma has not been previously described.

## Case presentation

An 85-year-old woman, noted to have been crouched on a street, was struck by a three-ton truck and was brought to the emergency department approximately 30 min after the accident. Upon arrival, her Glasgow Coma Scale score was E1V2M5; her heart rate was 117 bpm, her blood pressure was 120/99 mmHg, and her oxygen saturation was 99% on a 15-L non-rebreather mask. The initial focused assessment performed using sonography for trauma, including pericardial, perihepatic, perisplenic, and pelvic views, was negative. Although electrocardiography showed no significant changes, laboratory tests revealed a mildly elevated creatine kinase-MB level of 19.1 ng/mL and a markedly elevated high-sensitivity troponin I level of 1112.3 pg/mL. Contrast-enhanced computed tomography revealed a ruptured right CAA with active extravasation on the anterior aspect and a surrounding hematoma (Fig. 1). Additional injuries included a traumatic subarachnoid hemorrhage along the interhemispheric fissure, bilateral pulmonary contusions, left hemopneumothorax, fractures of the left 4th–8th ribs, right clavicle fracture, and left femoral shaft fracture with surrounding extravasation (Fig. 2, Fig. 3). Other injuries included right 7th–10th rib fractures, fractures of the C6 spinous process, L1–L4 transverse processes, a burst fracture of L2, right scapular fracture, active extravasation from the superior mesenteric artery branch, and hemorrhages in the descending mesocolon, right iliopsoas, and left gluteus maximus. Despite rapid transfusion and cardiopulmonary resuscitation, including intubation with mechanical ventilation and vasopressor support, the patient's consciousness progressively deteriorated. She died approximately 3 h after arrival at the emergency department.

**Fig. 1:**
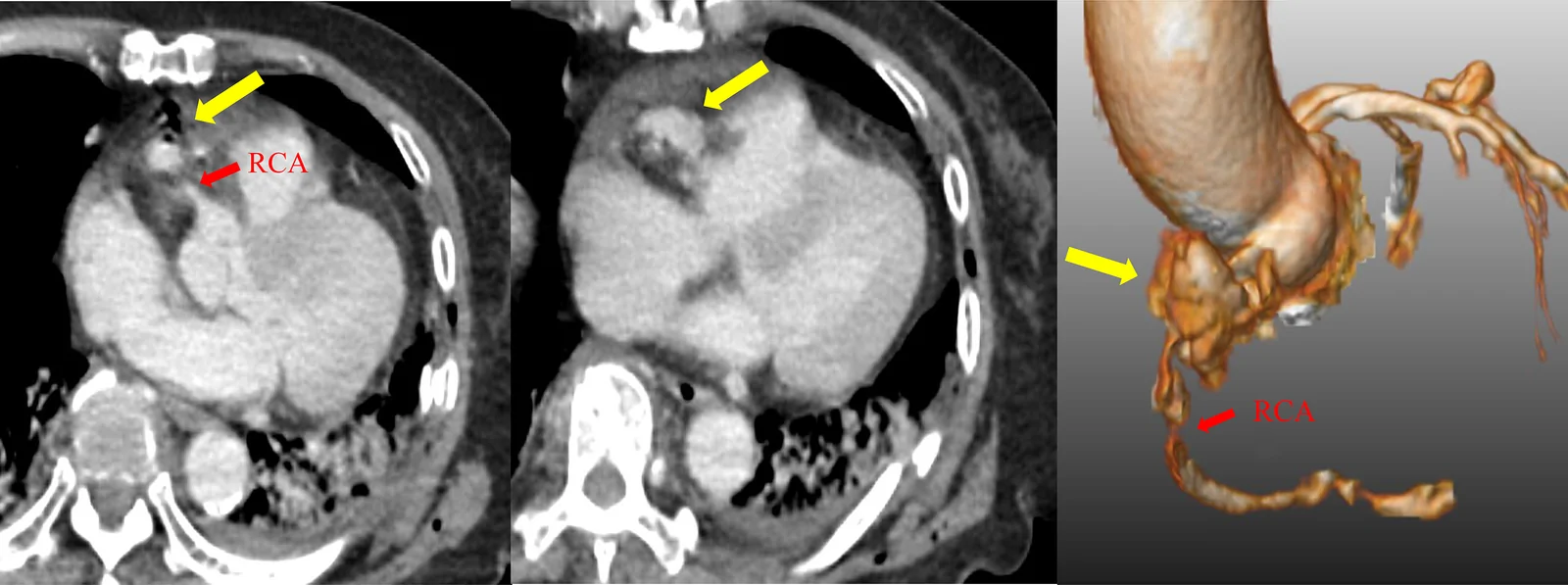
Extravasation is seen on the anterior aspect of the right coronary artery (RCA, red arrow) with a surrounding hematoma (yellow arrow).

**Fig. 2:**
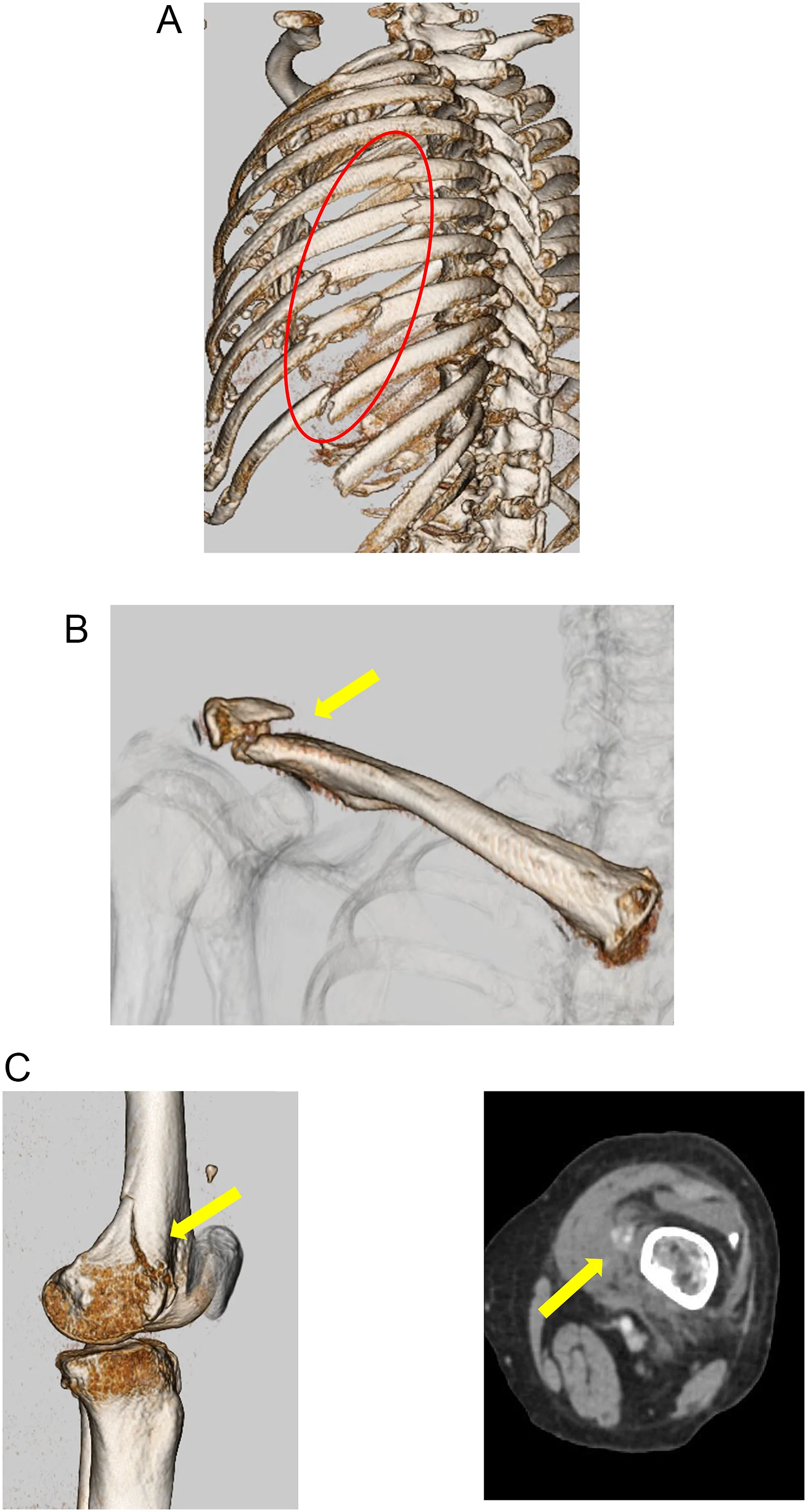
A: Fractures of the left 4th to 8th ribs (indicated by red ellipse). B: Right clavicle fracture (yellow arrow). C: Distal fracture of the left femur and surrounding extravasation (yellow arrow).

**Fig. 3:**
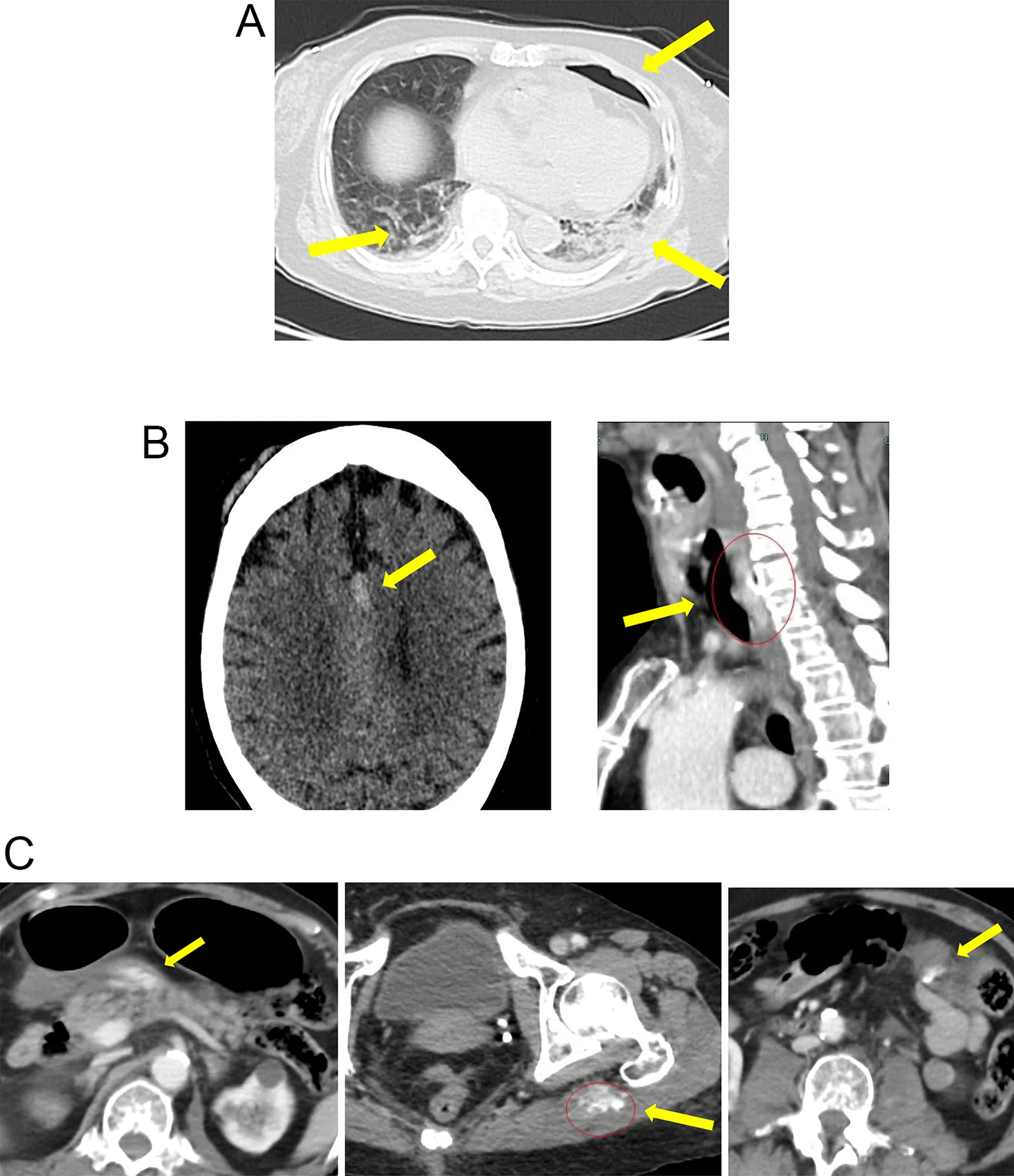
A: Bilateral pulmonary contusions and left hemopneumothorax (yellow arrow). B: Left: Traumatic subarachnoid hemorrhage in the interhemispheric fissure (yellow arrow). Right: Extravasation along the anterior aspect of the C6–C7 and T1 vertebral bodies (yellow arrow). (C, cervical; T, thoracic) C: Left: Hematoma and extravasation in the lesser sac and anterior surface of the pancreatic body (yellow arrow). Middle: Intramuscular hemorrhage within the left gluteus maximus (yellow arrow). Right: Extravasation within the transverse mesocolon (yellow arrow).

## Discussion

CAAs are defined as coronary dilatations that exceed the diameter of normal adjacent segments or that of the patient's largest coronary vessel by 1.5 times. They are typically asymptomatic and found incidentally. Aneurysmal dilatation has been reported in approximately 0.3%–5% of patients undergoing coronary angiography [1]. In terms of location, the right coronary artery is the most common site, followed by the left anterior descending artery, circumflex artery, and left main trunk. CAAs are primarily attributed to atherosclerosis, while other etiologies include congenital, inflammatory, infectious, and iatrogenic factors related to catheter intervention [2]. However, the natural history of CAAs and the incidence of complications, such as rupture or thrombosis leading to myocardial infarction, remain unclear.

Although rare, CAA rupture is a life-threatening event that may present as chest pain, infarction, or hemodynamic collapse. The reported cases often require emergency surgical intervention. The standard approach involves opening the aneurysm, suturing the afferent and efferent vessels, and performing coronary artery bypass grafting, if necessary. Endovascular options, such as coil embolization and covered stent placement, have also been described [3].

Here, we present a rare case of fatal trauma that likely occurred secondary to CAA rupture. The most challenging aspect of this case was determining the sequence of events. The critical detail that the patient was found crouched in apparent distress before the fatal trauma strongly suggests that the intense pain or hemodynamic collapse caused by the aneurysmal rupture was the primary event, leading to the patient's collapse, which subsequently resulted in the collision with the truck. The clinical findings further support the rupture-first sequence: laboratory tests revealed a markedly elevated high-sensitivity troponin I level of 1112.3 pg/mL, highly consistent with an acute primary cardiovascular event preceding the trauma. Furthermore, the definitive diagnosis of a ruptured RCA with active extravasation was obtained via the trauma screening computed tomography, confirming an ongoing vascular emergency. To the best of our knowledge, no previous report has described blunt trauma as directly causing CAA rupture, further reinforcing our interpretation.

This case provides crucial clinical lessons for emergency physicians. While traumatic cardiac injury, such as myocardial contusion or pericardial tamponade, must be ruled out in blunt trauma, the initial focused assessment using sonography for trauma was negative, and the cardiac biomarker elevation was disproportionately high. These factors highlight the potential diagnostic pitfall where severe external trauma masks an underlying internal catastrophe. This case emphasizes the critical importance of actively considering primary vascular emergencies as underlying causes in trauma patients who present with atypical findings or refractory shock.

## Conclusion

This case highlights the need to consider vascular emergencies in trauma settings, especially when early findings suggest a possible nontraumatic cause. Although trauma-induced CAA rupture cannot be excluded entirely, the sequence of clinical findings supports rupture as an initial event. To the best of our knowledge, no previous report has described blunt trauma as directly causing CAA rupture, further reinforcing our interpretation.

## CRediT authorship contribution statement

**Yoichi Hisata:** Conceptualization, Investigation, Visualization, Writing – original draft. **Takafumi Yamada:** Supervision, Writing – review & editing. **Akihiko Yamamoto:** Writing – review & editing. **Shunsuke Taguchi:** Writing – review & editing.

## Informed consent

Written informed consent was obtained from the patient's family for the publication of this case report and any accompanying images.

## Ethical approval

Institutional Review Board (IRB) approval is not required for case reports at our institution.

## Funding

This study did not receive any specific grants from funding agencies in the public, commercial, or nonprofit sectors.

## Declaration of competing interest

The authors declare that they have no known competing financial interests or personal relationships that could have appeared to influence the work reported in this paper.

## Data Availability

No datasets were generated or analyzed in the current study.
